# Bibliographical Notices

**Published:** 1841-01-09

**Authors:** 


### BIBLIOGRAPHICAL NOTICES.

*Introductory Lecture on Anatomy.* By Thomas Miller, M. D., Professor of Anatomy and Physiology, Columbian College, D. C. Delivered on Thursday, the 5th of November, 1840. Washington.

This is a well arranged, well written, and instructive lecture, portraying to the student, step by step, the obstacles which have already been surmounted in the pursuit of anatomy, captivating his attention by allusion to some of the most interesting points of physiology—exciting his ambition by exhibiting how much yet remains to be effected, and how completely it is within the power of each by industry to aid in its further advancement.

After a rapid survey of the wonders of organisation, its gradual complication in the ascent from the lower to the higher orders of beings, the almost imperceptible change from vegetable to animal life, rendering the line of separation equivocal, the Lecturer enters upon the study of man, and exhibits forcibly the necessity of frequent dissection as an essential element in its pursuit—not content with proving that the practice of medicine is dependant upon a knowledge of anatomy, which constitutes its only true basis, and showing *a fortiori* its importance to the surgeon, he maintains that the value of anatomy is not limited to our own profession, that it is necessary to the sculptor and painter, and useful to the jurist—he even asks, "if it is not to the interest of the artizan to understand the most completely constructed machine in existence?—to have a perfect model of mechanism?" He repels successfully the argument, that many practice efficiently without its assistance, and combats as superstitious the popular prejudice which has generally existed against dissections, dating a new era in medicine, from the permission to prosecute them, accorded by the Alexandrian school. These points are all ably illustrated, and we only regret that the limits of a weekly journal will not permit us to enter more fully into their detail.

*A Memoir of the Life and Character of the late Joseph Parrish, M. D.* By GEORGE B. WOOD, M. D.

THIS memoir is a just and beautiful tribute to the memory of departed excellence—the portraiture of a wise and virtuous life, executed with taste, fidelity, and feeling. To the profession of this community it is an acceptable, because a faithful sketch of one who was long honoured and respected among them, and it cannot fail to be perused with general interest as an elegant and attractive biography. The late Dr. Parrish was one of the oldest and most reputable practitioners of Philadelphia. Unconnected with any of its schools, his reputation was not much extended beyond the limits of the immediate sphere of his usefulness; but here he for a number of years occupied the first rank in the estimation of the community and his brethren. He was distinguished as an original thinker, an acute observer, and a sagacious, ready, and very successful practitioner. He was a popular teacher and lecturer, and the private instructer of a large number of the most respectable physicians of Philadelphia, among them, his biographer, who was a favourite pupil and intimate and confidential friend. In professional intercourse, Dr. Parrish was a model—free from jealousy or bitterness, undeviatingly straight-forward, scrupulous, and courteous. In private life admirable, he offered a rare combination of virtues and talents, and ran a career eminently useful and honourable, to which we who survive him cannot revert without pleasure and profit.

Dr. Parrish was born in the year 1779, of a respectable Quaker family. He had a strong natural bias for the study of medicine, for which he abandoned a mechanical employment in which his father had placed him. In his twenty-second year he entered the office of the late professor Wistar, and received his degree of doctor of medicine in the University of Pennsylvania in 1805. Aided by the patronage of the religious society of which he was a member, his professional skill and pleasing address rapidly brought him into a valuable practice, which he enjoyed for a long period, beloved by his patients, respected and consulted by his professional brethren. The career of Dr. Parrish was rather a useful than a brilliant one. His was a character valuable in its day, but not destined to exert a marked influence upon the future. In the language of his biographer,

"He was characterized by quick perception, an excellent memory for facts, and an unusual correctness of judgment. Little that he had the opportunity of hearing or seeing escaped his observation, and what he had once stored up in his mind was ever afterwards at his command. He had little imagination, and was without the taste and perhaps the ability for abstract and speculative reasoning, which too often busies itself in constructing edifices of conclusion upon slender premises, and wastes in vain attempts to establish general truths the time which would be better spent in collecting facts. But he was gifted, in an extraordinary degree, with that practical faculty which turns to useful account whatever comes within its reach; which, by a sort of intuition, distinguishes a truth amidst the rubbish by which it is concealed, and out of a labyrinth of conflicting means selects that which most surely leads to the end in view. His was, indeed, eminently a practical mind, looking always to acts rather than to opinions, and disposed to measure the value of any system or project by its probable bearing on the condition of society or individuals, not by its mere beauty, or the ingenuity displayed in its invention."

As a practitioner, Dr. Parrish was original and eminently successful. In many diseases, his plan of treatment was in decided opposition to the prevailing fashions of the day. In pulmonary consumption he abandoned the antiphlogistic for the opposite system of generous living and vigorous exercise in the open air—and with the happiest results. Shortly after his entrance into business,

"The great typhus epidemic, which so long scourged this country, made its appearance in Philadelphia. At its first appearance, this complaint was not fully understood. Physicians were not generally prepared to recognise a disease of debility associated with apparently violent inflammation, and were in the beginning too apt to overlook the tendency to prostration which lurked fatally beneath the show of excitement. The attention of Dr. Parrish had been strongly directed to the subject by the perusal of a treatise by Dr. North, who had seen much of the disease in New England, and who strenuously advocated the stimulant treatment. His aversion to theory in medicine left him open to the evidence of facts, however opposed to prevailing opinions; and he was quite prepared to encounter the disease by methods which had stood the test of experience, rather than by those which analogy alone would appear to indicate. The epidemic approached Philadelphia through New Jersey, and hung for a while over the opposite shore of the Delaware, before it burst upon our city. The inhabitants were alarmed by reports of a terrible disease in the town of Camden, which appeared to bid defiance to medicine. Dr. Parrish was called in to the aid of the physicians of the neighbourhood. At the period of his first visit, seven cases had occurred, and all proved fatal. He was told that the disease was of an inflammatory nature, and had been treated by the lancet and other depletory measures. Its malignant aspect at once struck his attention. He saw through the veil of inflammation which it had thrown over its ghastly features, and beheld the deadly weakness beneath it. He advised an immediate abandonment of the lancet, and the substitution of an actively stimulant treatment. The effects were most happy. Numbers now got well where before all had died. A disease supposed to be almost incurable was found to be, in the great majority of cases, under the control of medicine. The terrors of the first awful reports gave way before the happier intelligence which followed; and the newly inspired confidence was directed especially towards the author of the change. When the epidemic reached the city, Dr. Parrish found himself in the midst of an ample business; and the devotion which he paid to the sick, and the skill and success which marked his efforts, gave him a place in the opinions and affections of his fellow-citizens which he did not lose when the immediate occasion ceased. His views of the disease and its treatment met with much opposition; and some decision of character was required to carry them into effect. On one occasion, a physician in attendance with him upon two cases of the disease in the same family, believing them to be highly inflammatory, strongly urged the employment of the lancet, and upon being resisted by Dr. Parrish, who felt convinced that the proposed remedy would be fatal, retired from attendance, leaving the whole responsibility with his colleague. The ground of difference was known, and the eyes of the whole neighbourhood were directed with intense expectation towards the result. 'You cannot conceive,' said Dr. Parrish, in relating the circumstance to his pupils, 'the anxiety I experienced.' Happily, however, both patients recovered, and the event contributed to extend his reputation."

Like his cotemporary, Physick, Dr. Parrish was distinguished as a surgeon as well as a physician. For several years he filled the posts of surgeon to the Philadelphia Alms House and Pennsylvania Hospital, and was highly thought of, for judgment and operative skill.

Dr. Parrish closed a long and honourable career, in March, 1840, meeting death with the calmness of a philosopher and resignation of a Christian. A considerable fortune was the result of his professional accumulation. Better than these, he left behind him an unstained reputation, and a memory, which is cherished for the purity, generosity, and single-heartedness of his character. Many traits illustrative of these amiable qualities are recorded by Dr. Wood. From them, we select the following:

"A striking instance of the influence of a sense of duty over his conduct, was in his declining to take the office of Professor of Anatomy in the University of Pennsylvania, which he believed, and I have no doubt upon the best grounds, to have been at one time within his reach. I have said that he was naturally fond of distinction; and this was a post to which he believed himself competent, and in which he would probably have attained much credit and a wide-spread popularity. An ordinary person, in his situation, would have seized upon it with avidity. But he regulated his conduct by a higher standard than that of personal gratification. He believed that a station in the University would bring what might be considered his duty towards the Institution into frequent conflict with his peculiar religious sentiments and habits. He was unwilling to expose himself to temptations, likely to loosen his hold upon those principles which he conceived to be the anchor of his safety. To his intimate friends, who urged him to avail himself of this opportunity, he was wont to answer, in his naive and cheerful but impressive manner, by pointing to his breast, and observing that he wished to have all comfortable there; that no worldly advantages would be any compensation for the loss of that heart-felt satisfaction which attended obedience to the intimations of his inward monitor. This was, indeed, the great rule of his life. Believing most fully in that fundamental Quaker doctrine that the Divine Spirit communicates directly with men, that from this source is the 'true light which lighteth every man that cometh into the world,' and that consequently every individual has a sure counsellor in his own breast, which, if consulted in the right spirit, will never fail or mislead him; he was in the constant habit of looking inward for intimations of duty, and of submitting to them implicitly, however opposed to his apparent worldly interests. Now, whatever opinion may be entertained of these intimations, whether we agree with the Friends in considering them as of supernatural origin, or believe them, as most men do, to proceed from the natural workings of the mind, under the influence of education, habit, reason, and conscience, it is nevertheless the fact that, in any case of morals, an individual, brought up in a civilized and Christian country, will seldom go far astray, who uniformly consults them with a single eye to the truth. Dr. Parrish believed that he found peace and safety in this rule of action: and no merely worldly temptation was strong enough to remove him from any position which he had taken in conformity with it. The same motives which induced him to forego the opportunity of obtaining a professorship in the University, caused him also to decline offers, and resist solicitations afterwards made to him to join other incorporated medical schools. 'My bark,' he used to say, 'was made for quiet waters.'"

Dr. Wood thus beautifully sums up his sketch of this truly admirable character:

"Firmness and courage were also among the moral qualities which distinguished Dr. Parrish. With all his kindness of heart and disposition to please, though no man was less tenacious of opinion for opinion's sake, and none more disposed to yield in trifles to the convenience or even caprice of others, yet in all affairs which involved a point of principle he was immoveable, and did not hesitate to do or to avow what he believed to be his duty, whatever personal injury or odium might accrue.

"Thus morally courageous, he was not wanting in that less noble attribute which leads to contempt of danger. During an intimate intercourse of many years, I do not remember to have seen him, in any one instance, exhibit the least evidence of bodily fear. In pestilence he was among the foremost at the post of danger. During the prevalence of yellow fever, I have seen him by day and by night, without the expectation of pecuniary recompense, and at a period of his professional life when he had nothing further to wish for on the score of reputation, enter the deserted precincts of infection, and expose himself to the most imminent danger, in attendance upon individuals who had been seized by the disease while lingering behind the fleeing population. On the bed of sickness and death, with a clear knowledge of his danger, he was quite composed, and never exhibited any of those fearful apprehensions which sometimes beset the closing scenes even of those best prepared to die. Such, indeed, was his natural temperament, that danger attended with the opportunity for exertion, seemed to have charms for him; and I have heard him more than once say, not in a boastful spirit, but quite naturally, as if merely giving expression to the feelings of the moment, that, were he not opposed on principle to all wars and fightings, he should take a stern delight, in a cause which he could approve, in leading the forlorn hope of an assault."

"But, while thus marked with striking traits, he was not without the graces also of character. His amiableness of temper, candour and openness of heart, liberality of sentiment, charity for the failings of others, warmth and constancy in friendship, and love of order and punctuality, were often beautifully illustrated in his daily intercourse, and contributed to give him the charm of manner which rendered his presence every where so acceptable. The real politeness for which Dr. Parrish was remarkable, was in no respect the result of cultivation, but flowed directly from the fountain of his own kindly feelings. It was the genuine coinage of nature, which art may counterfeit, but seldom equals. With a self-possession resulting from his utter want of pretension and the perfect simplicity of his character, and entirely free from that sort of diffidence of manner which is the frequent result of pride, he was never awkward in speech or movement, and in all the intercourse of life exhibited the deportment of a true gentlemen."

"The almost unprecedented array of his fellow citizens of all classes who attended his remains to the grave, the general expression of regret for his loss, and the measures taken by the various bodies to which he belonged, to procure some public commemoration of his worth and services, are evidences of a general esteem and affection such as seldom fall to the lot of individuals unconnected with public life. Perhaps no one was personally known more extensively in the city, or had connected himself by a greater variety of beneficent service with every ramification of society. It is true that no marble has been erected over his remains, and that the very spot where they are laid, will soon be undistinguishable to every eye save that of conjugal or of filial love; yet the remembrance which he has left behind him, the only monument which the rules of his unostentatious sect allow, is far more precious than the praises of carved stone, which gold may purchase or power command."

